# Epicardial Adipose Tissue in Obese Heart Failure With Preserved Ejection Fraction: The Evidence Grows∗

**DOI:** 10.1016/j.jacadv.2023.100731

**Published:** 2023-11-22

**Authors:** Masaru Obokata, Kazuki Kagami

**Affiliations:** a Department of Cardiovascular Medicine, Gunma University Graduate School of Medicine, Maebashi, Gunma, Japan; b Division of Cardiovascular Medicine, National Defense Medical College, Tokorozawa, Saitama, Japan

**Keywords:** epicardial adipose tissue, heart failure with preserved ejection fraction, obesity, right ventricle, ventricular interdependence

**Figure:**
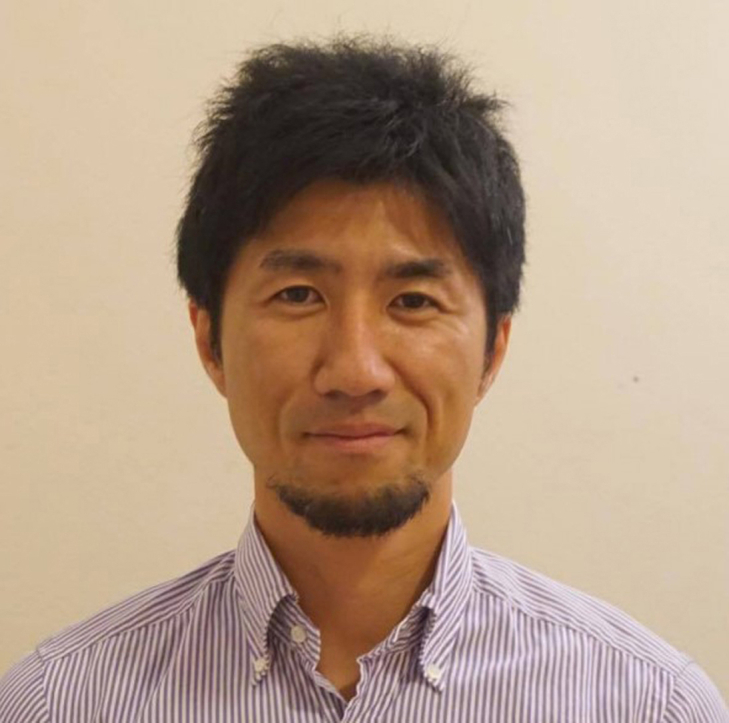

Obesity has reached epidemic proportions in most of the westernized countries; currently, 40% of adults in the United States are obese. It is projected that 1 in 2 American adults will be obese by 2030.^1^ Obesity is substantially common in patients with heart failure and preserved ejection fraction (HFpEF). Recent data have changed our thinking about the impact of obesity and increased adiposity on HFpEF—from a cause of dyspnea or mere comorbidity to the primary cause of HFpEF or an important modifier of its pathophysiology.^2^, ^3^, ^4^ It is now well understood that the contribution of obesity to HFpEF is multifactorial, including cardiac remodeling, myocardial dysfunction, abnormal cardiac metabolism and energetics, volume overload, pericardial restraint, and skeletal muscle abnormalities through its hemodynamic, inflammatory, mechanical, and neurohormonal effects.^1^^,^^5^

Recent interests have focused on epicardial adipose tissue (EAT) in the pathophysiology of HFpEF.^2^^,^^3^^,^^6^, ^7^, ^8^ EAT is located directly adjacent to the myocardium. In a healthy state, EAT has been speculated to be involved in cardiac energy regulation or to mechanically protect myocardium and coronary arteries. With excessive accumulation, however, EAT may have multiple detrimental effects on the heart. Previous studies have demonstrated that EAT is increased in patients with HFpEF compared to non-heart failure controls and in obese HFpEF relative to nonobese HFpEF.^2^^,^^3^^,^^6^ Increased EAT in HFpEF is reported to be associated with elevated biventricular filling pressures, enhanced ventricular interdependence, myocardial fibrosis, exercise intolerance, and an increased risk of adverse clinical outcomes.^3^^,^^6^, ^7^, ^8^ Despite these compelling data and the fact that 75% of EAT is located on the surface of the right ventricle (RV), the relationship between EAT and RV dilatation and its impact on clinical outcomes in patients with HFpEF is poorly understood.

In this issue of the *JACC: Advances*, Nakamori et al^9^ present new data regarding pathophysiologic and prognostic impact of EAT volume and RV dilatation in patients with HFpEF. The authors conducted a retrospective cohort study of 77 nonobese and 73 obese patients with HFpEF who underwent cardiac magnetic resonance (CMR) imaging. CMR images were analyzed to measure EAT volume, total RV volume (TRVV, the sum of RV end-diastolic volume and RV mass), total heart volume (the sum of TRVV, left ventricular [LV] end-diastolic volume, LV mass, and EAT volume), RV systolic function (RV free wall longitudinal strain), and ventricular interdependence by the LV eccentric index. Echocardiography was also performed to assess RV afterload (ie, estimated RV systolic pressure).

The authors found that EAT volume, TRVV, and total heart volume were larger in patients with obese HFpEF than those in nonobese HFpEF, while LV eccentricity index did not differ between the groups. Notably, the sum of EAT volume and TRVV was associated with LV eccentric index, and this trend appeared more pronounced in patients with obese HFpEF (r = 0.36, *P* = 0.002). After a median follow-up duration of 46 months, there were 91 all-cause deaths and heart failure hospitalization (47%). Consistent with prior studies,^7^^,^^8^ larger EAT was associated with higher rates of adverse clinical outcomes regardless of obesity status. The authors further demonstrated that obese patients with larger EAT (≥130 mL) and TRVV (≥180 mL) had an increased risk of the composite outcome.

The authors are to be commended on this important contribution, which confirms and extends upon earlier studies while providing important new insights regarding the impact of EAT and RV dilatation in patients with obese HFpEF. The most important finding is the association between EAT volume and clinical outcomes in HFpEF, consistent with 2 previous studies, 1 evaluating EAT by echocardiography and the other by CMR.^7^^,^^8^ What are the possible mechanisms of this relationship? The adverse effects of increased EAT in HFpEF would be multifactorial. Although the strength of the correlation in the current study was modest, EAT was associated with lower RV free wall strain and larger TRVV. This suggests that EAT accumulation may contribute to RV dysfunction, dilatation, and remodeling, which are associated with poor prognosis in HFpEF.^10^ This is likely related to the production of pro-inflammatory cytokines and pro-atherogenic adipokines by dysfunctional EAT or the infiltration of adipose tissue into the adjacent myocardium.^8^

Consistent with a prior study showing the association between EAT thickness and LV eccentricity index in obese HFpEF,^3^ the current study demonstrated that the sum of EAT volume and TRVV was associated with LV eccentricity index in patients with HFpEF, especially in those with obese HFpEF. This extends the prior study and supports a potential role of EAT in pericardial restraint and enhanced ventricular interdependence, especially in the setting of RV dilatation. Obesity is associated not only with increased EAT but also with RV dilatation, possibly due to plasma volume expansion, abnormal venous compliance and capacitance, and sodium retention by activation of the sympathetic nervous system and the renin-angiotensin-aldosterone system.^1^^,^^2^^,^^4^^,^^5^ The observed association between larger EAT and TRVV and higher rates of clinical outcomes in obese HFpEF indicates that worsening ventricular interdependence may mediate this relationship.

Regardless of the mechanisms, the current findings and others suggest that EAT is a potential therapeutic target or, at least, that increased EAT is a useful surrogate that could be used to identify patients at increased risk. There may be 3 ways to target EAT: reduce the amount of EAT, suppress pro-inflammatory cytokines produced by EAT, and reduce pericardial restraint. Potential therapies specifically designed for reducing EAT volume may include body weight reduction by aerobic exercise, diet, pharmacotherapy, or bariatric surgery, sodium-glucose co-transporter 2 inhibitors, and glucagon-like peptide 1 receptor agonists.^11^, ^12^, ^13^, ^14^, ^15^ Bariatric surgery leads to dramatic and sustained weight reduction, and 1 observational study indicates marked reduction in EAT.^13^ Sodium-glucose co-transporter 2 inhibitors and statins may potentially ameliorate inflammation in EAT.^16^^,^^17^ Surgical or minimally invasive pericardiotomy may be effective in reducing pericardial restraint in patients with obese HFpEF.^18^

In summary, Nakamori et al.^9^ have provided new data regarding the pathophysiologic and prognostic impact of EAT and RV dilatation in patients with obese HFpEF. What is needed now is further study to determine if increased EAT is a therapeutic target and, if so, to explore the best treatment options.

## Funding support and author disclosures

Dr Obokata has received research grants from the Fukuda Foundation for Medical Technology, Mochida Memorial Foundation for Medical and Pharmaceutical Research, Nippon Shinyaku, Takeda Science Foundation, Japanese Circulation Society, Japanese College of Cardiology, AMI Inc, Nippon Boehringer-Ingelheim, JSPS KAKENHI (21K16078), and AMED (23jm0210104h0002); and has received speaker honoraria from Novartis, Otsuka Pharmaceutical, Eli Lilly, AstraZeneca, and Nippon Boehringer-Ingelheim. Dr Kagami has reported that he has no relationships relevant to the contents of this paper to disclose.
